# Embolisation of Posttraumatic Superior Mesenteric Artery Pseudoaneurysm in a Patient with Short Bowel Syndrome Preceding Bowel Transplantation

**DOI:** 10.1155/2011/539340

**Published:** 2011-07-28

**Authors:** Vinko Vidjak, Karlo Novacic, Marko Slavica

**Affiliations:** Department of Diagnostic and Interventional Radiology, Clinical Hospital Merkur, Zajceva 19, 10000 Zagreb, Croatia

## Abstract

Penetrating abdominal trauma often causes bowel injuries which may lead to “short bowel syndrome” which is a potential indication for bowel transplantation. Posttraumatic pseudoaneurysms of abdominal arteries are often a result of penetrating abdominal trauma. We report a successful embolisation of posttraumatic superior mesenteric artery (SMA) branch pseudoaneurysm using microcoil, in a patient with short bowel syndrome who was successfully transplanted three months after embolisation.

## 1. Case Report

**Figure 1:**
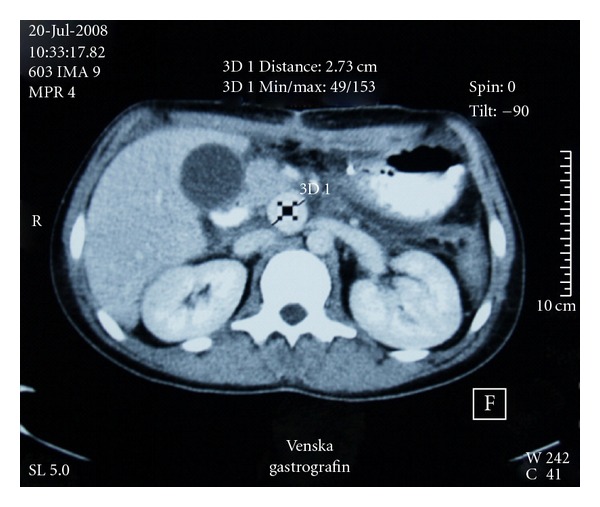
25-years-old male patient with SMA pseudoaneurysm. Transverse CT scan in delayed venous phase (45 seconds), slice thickness showing oval hyper vascular lesion between aorta and inferior vena cava 27 mm in diameter.

**Figure 2:**
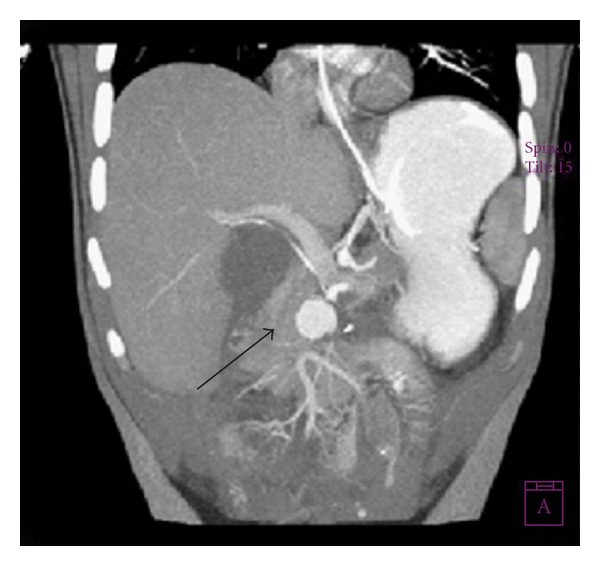
Coronal MPR MSCT angiography showing pseudoaneurysm and occlusion of part of SMA.

An 18-year-old male patient was brought to local county hospital in July 2008 with multiple stab wounds in the abdominal region. Due to severe blood loss and life-threatening conditions, he was submitted to several surgery procedures, resulting in major resection of large and small bowel segments. Because of his general poor condition and limited surgical options in the county hospital, the patient was transferred to our institution 20 days after for further treatment. During first 24 hours, his general condition was stable. Ultrasonography (US) with color Doppler (CD) showed a hypoechoic lesion with vascularisation and typical “to and fro” Doppler flow pattern which is characteristic for pseudoaneurysm (PSAN). Multislice computed tomography (MSCT) angiography of the abdomen revealed a rounded hyperdense lesion 27 mm in diameter filled with contrast medium in early arterial phase of imaging, and this confirmed the diagnosis of pseudoaneurysms located next to the ostium of superior mesenteric artery (SMA) (Figures 1 and 2).

Short bowel syndrome is described as a severe condition which requires intestine transplantation; however, in our case, unavailability of an immediate intestine donor and uncertain time frame for bowel transplantation and simultaneous open surgery pseudoaneurysm repair led to the decision to perform a digital subtraction angiography (DSA) and endovascular treatment (embolisation) of PSAN.

**Figure 3:**
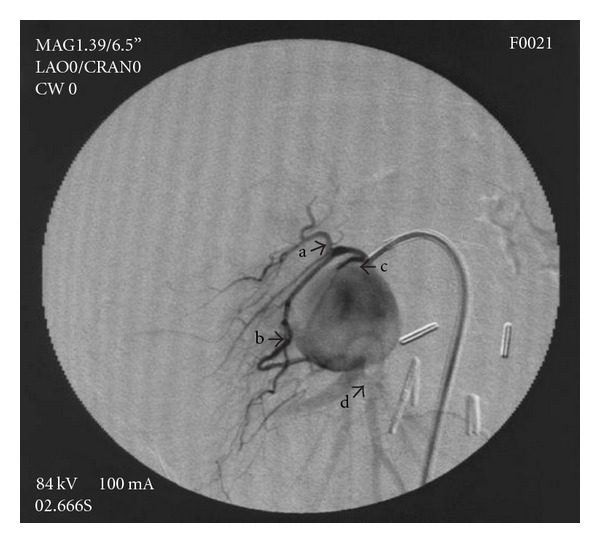
Selective angiography of SMA showing pseudoaneurysm, occlusion of short part of SMA and patent SMA below pseudoaneurysms, (a) inferior pancreaticoduodenal artery, (b) inferior pancreaticoduodenal artery branches, (c) short neck of artery filling pseudoaneurysm, and (d) distal part of SMA.

**Figure 4:**
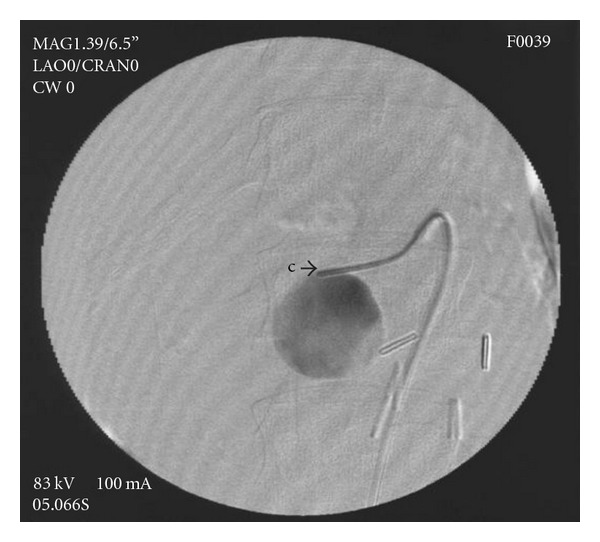
Superselective angiography through the feeding artery (aneurysmography), showing no vessels arising from pseudoaneurysms.

DSA of celiac trunk and SMA were preformed from a right common femoral artery (CFA) approach, using a 5F Cobra diagnostic catheter (Terumo Europe N.V. Leuven, Belgium) and Ultravist 370 (Bayer HealthCare Pharmaceutical Inc.) contrast agent. Selective DSA of the celiac trunk showed no pathological findings. Selective SMA angiography showed the replaced right hepatic lobe artery originating from SMA. DSA of the SMA distal to origin of replaced right HA revealed an pseudoaneurysm filling from branch of inferior pancreaticoduodenal artery, occlusion of main SMA trunk at the level of pseudoaneurysm, and collateral filling with patent SMA distal to pseudoaneurysm (Figure 3). Superselective angiogram of branch of the inferior pancreaticoduodenal artery filling the pseudoaneurysm showed no communication with other arteries or extravasation in peritoneal space (Figure 4). 

**Figure 5:**
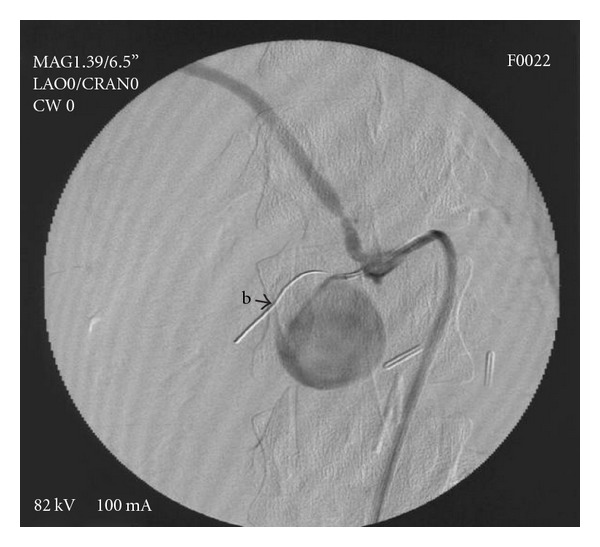
(b) “safe” guide wire in the main trunk of inferior pancreaticoduodenal artery, visualisation of replaced right hepatic artery, and filling of pseudoaneurysm.

**Figure 6:**
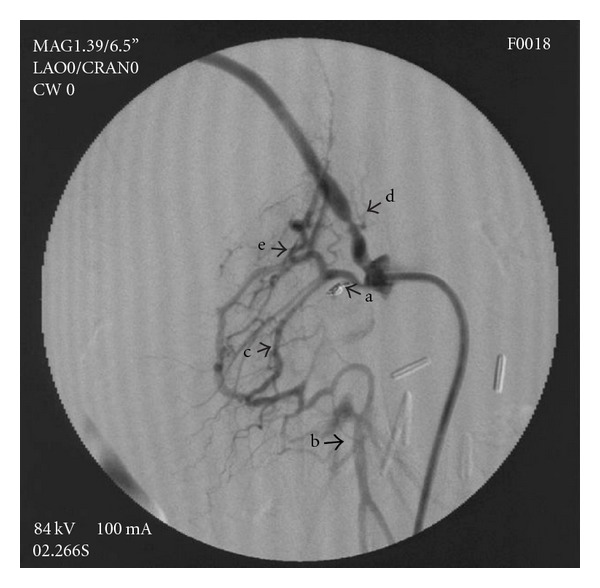
postembolisation angiogram showing complete exclusion of pseudoaneurysm without contrast filling. (a) coils within lumen, (b) postocclusive SMA, (c) patent inferior pancreaticoduodenal artery, (d) replaced right hepatic artery, and (e) branches of inferior pancreaticoduodenal artery.

Upon these finding, we decided to embolise the feeding artery with coil (or multiple coils if needed). We used a guiding catheter (CBL IG 8Fr, Cordis Corporation, Miami Lakes, Fla, USA, USA), microcatheter (Rapid transit 2.8Fr, Cordis Europe N.V. Netherlands), and additional “safe” 0.014*″* guide wire positioned in the main tree of inferior pancreaticoduodenal artery, allowing for additional stabilization of the system and to have wire in place in case of inadvertent embolisation (Figure 5). The tip of microcatheter was positioned in the neck of the pseudoaneurysm (2 mm width, 5 mm length), and 2 pushable microcoils (Trufill Cordis Neurovascular, Miami, Fla, USA, USA) 3 mm × 40 mm were inserted in the pseudoaneurysm's neck. Follow-up DSA showed total occlusion of the pseudoaneurysm's neck without the need for additional coils and with the preserved blood flow in the main trunk of inferior pancreaticoduodenal artery (Figure 6).

**Figure 7:**
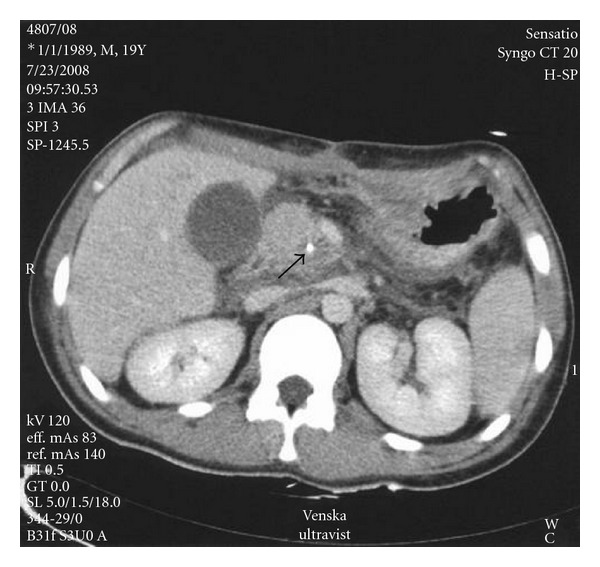
CT scan 24 hours post embolisation in delayed venous phase (45 sec), collimation 5 mm showing complete thrombosis of pseudoaneurysm. Hyperdense small round object (arrow) consistent with coils.

Follow-up USCD and MSCT 24 hrs later showed a complete thrombosis of SMA pseudoaneurysm (Figure 7). Three months later (November 2008), the patient was submitted in our institution, a prominent center for solid organs transplantation, for intestinal transplantation. He obtained normal intestinal function and was discharged on house care in stable condition but died, 9 months later, from severe infection due to immunodeficiency.

## 2. Discussion

Penetrating abdominal trauma often causes visceral arteries lesions, with possible pseudoaneurysm formation as a consequence of arterial wall disruption and formation of a local haematoma bordered by fibrous tissue [1–4]. In contrast to true aneurysms which involve all three layers of the arterial wall, pseudoaneurysms are false aneurysms, which consist of a cavity filled with blood, located between the artery and overlaying connective tissue, communicating with the arterial lumen. The superior mesenteric artery SMA pseudoaneurysms, unlike other visceral artery pseudoaneurysms, often cause clinical symptoms and demand in-time intervention [5]. There are several minimal invasive (“no open surgery repair”) approaches which result in lower mortality versus open surgery approach (15%–40%) especially in patients with severe clinical status [6–13]. Large posttraumatic bowel resections cause short bowel syndrome, which is an indication for bowel transplantation [14].

Abdominal penetrating trauma can lead to hemorrhagic shock and ischemic colitis; however, posttraumatic gangrenous bowel lesions are rarely seen in younger people [15]. Extensive bowel resection (>50% short bowel length) presents a risk for developing short bowel syndrome. Bowel transplant is indicated in stable patients with remaining (after resection) small bowel shorter than 50 cm and removed colon [14]. Hence, bowel transplant was the final solution because of the grave clinical state of our patient and it depended on the availability of a donor organ. Initially, the simultaneous procedure with surgical repair of PSAN and bowel transplantation was planned but, due to the uncertainty of the donor organ availability and possible complication of PSAN rupture, it was decided to proceed with pseudoaneurysm embolisation to prevent any possible complications and deterioration of the patient's general condition [5]. Thus, we managed to avoid an additional laparotomy before the final one-bowel transplant.

Abdominal visceral artery aneurysm incidence is 0.01–2% where the SMA aneurysm is the third most common (5%) [6]. Penetrating trauma is a significant factor in pseudoaneurysm formation [2, 3]. The danger inherent in these pathological lesions is their potential to rupture (38%) [13]. During the past several decades, the available methods for the treatment of aneurysmal/pseudoaneurysmal lesions included surgical bypass and ligatures, which were accompanied by high mortality rates (up to 56% in emergency cases and up to 10% in elective patients) as compared to minimally invasive methods (mortality almost 0% with morbidity up to 20% in emergency patients) [7, 16]. Current treatment options consist of minimally invasive techniques: laparoscopic operations and endovascular or percutaneous embolisations. For percutaneous vascular occlusive procedures, a variety of devices and agents can be used: metal coils, detachable coils, gelfoam, PVA, thrombin, onyx, n-butyl cyanoacrylate (NBCA), stent grafts, and the combination of stent with coils [7–10, 16–18]. The prerequisites for a safe endovascular embolisation involve the stable position of the tip of the catheter in the target artery, clear view of the afferent and possibly efferent pseudoaneurysm arteries, the relationship of the targeted artery and adjacent arteries to minimize the risk of inadvertently embolisations, and the appearance of the pseudoaneurysm “wall,” especially because of a possible percutaneous embolisation (i.e., thrombin) [17]. When selecting the technique and embolisation materials, we considered important the fact that the pseudoaneurysm feeding artery is in very close contact with the important collateral arteries (right hepatic artery, inferior pancreaticoduodenal artery, and right and middle colic). This proximity prevented us from using the “sandwich technique” (isolation of PSAN) with coils or the use of liquid embolisation materials, and stent-graft as a method of treatment [10, 11, 19]. Although this was the case of a tight neck pseudoaneurysm, we did not rule out percutaneous pseudoaneurysm embolisation with NBCA with simultaneous neck occlusion by balloon catheter, which has been described in the literature [18]. However, the latter procedure was not our first method of choice. By wire manipulation inside the microcatheter and injection of contrast material under maximum possible pressure, we were assured that the tip of the catheter was safely positioned in the neck of the pseudoaneurysm. Aneurysmography showed that this was the case of an isolated pseudoaneurysm which had no communication with other arteries (Figure 3). By this way, the choice of the embolisation material was reduced to coils. During our previous embolisation procedures, we had more experience with using coils, compared with the other embolisation materials mentioned before. Considering the dimensions of the pseudoaneurysm neck (width 2 mm, length 5 mm), microcoil imposed itself as the best choice. Although the occlusion of a large aneurysm/pseudoaneurysm by a larger number of coils has been suggested, our patient's example shows that the embolisation of the pseudoaneurysm with a tight neck with two microcoils was sufficient.

Patient withstood the procedure well and had bowel transplantation three months later, living on supplementary nutrition in the meantime. However, 7 months after the transplantation, the patient's condition deteriorated (advanced infection with immunodeficiency) and he died 9 months after the transplantation or 12 months after the pseudoaneurysm embolisation.

Reviewing the available literature databases and according to our present knowledge, this is the first case of successful pseudoaneurysm embolisation of the SMA branch in a patient who had successful bowel transplantation due to short bowel syndrome.

##  Authors' Contribution

Dr. V. Vidjak and myself (Dr. K. Novacic) treated this patient together in cath lab, he as the primary operator and me as an assistant. Dr. M. Slavica was the second assistant during the procedure.
